# Effects of a nurse-based case management compared to usual care among aged patients with myocardial infarction: results from the randomized controlled KORINNA study

**DOI:** 10.1186/1471-2318-13-115

**Published:** 2013-10-29

**Authors:** Christa Meisinger, Björn Stollenwerk, Inge Kirchberger, Hildegard Seidl, Rupert Wende, Bernhard Kuch, Rolf Holle

**Affiliations:** 1 Institute of Epidemiology II, Helmholtz Zentrum München, German Research Center for Environmental Health, Neuherberg, Germany; 2 KORA Myocardial Infarction Registry, Central Hospital of Augsburg, Augsburg, Germany; 3 Institute of Health Economics and Health Care Management, Helmholtz Zentrum München, German Research Center for Environmental Health, Neuherberg, Germany; 4 Department of Internal Medicine I - Cardiology, Central Hospital of Augsburg, Augsburg, Germany; 5 Donau-Ries-Kliniken, Department for Internal Medicine/Cardiology, Nördlingen, Germany

**Keywords:** Elderly, Randomized controlled trial, Case management, Myocardial infarction

## Abstract

**Background:**

Transition from hospital to home is a critical period for older persons with acute myocardial infarction (AMI). Home-based secondary prevention programs led by nurses have been proposed to facilitate the patients’ adjustment to AMI after discharge. The objective of this study was to evaluate the effects of a nurse-based case management for elderly patients discharged after an AMI from a tertiary care hospital.

**Methods:**

In a single-centre randomized two-armed parallel group trial of patients aged 65 years and older hospitalized with an AMI between September 2008 and May 2010 in the Hospital of Augsburg, Germany, patients were randomly assigned to a case management or a control group receiving usual care. The case-management intervention consisted of a nurse-based follow-up for one year including home visits and telephone calls. Key elements of the intervention were to detect problems or risks and to give advice regarding a wide range of aspects of disease management (e.g. nutrition, medication). Primary study endpoint was time to first unplanned readmission or death. Block randomization per telephone call to a biostatistical center, where the randomization list was kept, was performed. Persons who assessed one-year outcomes and validated readmission data were blinded. Statistical analysis was based on the intention-to-treat approach and included Cox Proportional Hazards models.

**Results:**

Three hundred forty patients were allocated to receive case-management (n=168) or usual care (n=172). The analysis is based on 329 patients (intervention group: n=161; control group: n=168). Of these, 62% were men, mean age was 75.4 years, and 47.1% had at least either diabetes or chronic heart failure as a major comorbidity. The mean follow-up time for the intervention group was 273.6 days, and for the control group it was 320.6 days. During one year, in the intervention group there were 57 first unplanned readmissions and 5 deaths, while the control group had 75 first unplanned readmissions and 3 deaths. With respect to the endpoint there was no significant effect of the case management program after one year (Hazard Ratio 1.01, 95% confidence interval 0.72-1.41). This was also the case among subgroups according to sex, diabetes, living alone, and comorbidities.

**Conclusions:**

A nurse-based management among elderly patients with AMI had no significant influence on the rate of first unplanned readmissions or death during a one-year follow-up. A possible long-term influence should be investigated by further studies.

**Clinical trial registration:**

ISRCTN02893746

## Background

The population in Germany, as in many developed countries, is rapidly ageing [1]. While in 1950, there were 205 million persons aged 60 or over throughout the world, the global population aged 60 years or over is projected to expand by more than three times to reach nearly 2 billion in 2050 [1].

Ischemic heart disease is the leading cause of death among patients in Europe and other regions of the world [2] and most of the people who die of ischemic heart disease are 65 years and older [3]. Patients with myocardial infarction who were discharged alive still are at risk for postdischarge hospital readmission. Rates for readmission within 30 days considerably vary among countries with highest rates observed in the United States (39.1%) and 3.5% reported from Germany [4]. Older age was found to be a significant predictor of early readmission [5] and a 1-year readmission rate of 38% in patients > 65 years was reported by Andres et al. [6].

These trends result in a substantial financial burden on the health care system. Due to the aging of the population and the improved survival of patients with coronary heart disease (CHD) a large population of older adults is eligible for secondary prevention [7].

Guidelines for secondary prevention suggest lifestyle changes such as smoking cessation, increase of physical activity, weight management, risk factor control including blood pressure control, lipid and diabetes management, and pharmacological treatment for all persons with CHD [8]. There is increasing evidence that elderly men and women with CHD also benefit from secondary prevention measures [9]. However, elderly persons are more likely to have specific characteristics which may complicate the implementation of secondary prevention measures, e.g. the adherence to prescribed medication, such as reduced social support, multimorbidity, functional or cognitive impairments.

Secondary prevention after hospital discharge in Germany is provided by a number of actors within the health care system, specifically by general health practitioners and cardiologists. Around 50% of the German patients with an AMI receive an in-hospital cardiac rehabilitation over a period of 3 weeks in a specialized rehabilitation hospital. However, data suggest that patients aged above 60 receive in-hospital cardiac rehabilitation less often than younger persons [10]. Moreover, in Germany no home-based early post-discharge programs are available as compared to other countries [11]. Long-term disease-management programs for patients with CHD are offered by health insurance companies.

A number of intervention trials, mostly including persons younger than 70 years, investigated whether a post-discharge nurse-based case management may influence patient readmission and other outcomes in CHD [12,13]. Although the results of prior studies showed a positive effect on the process of care, survival, and functional status or quality of life of patients with CHD these findings cannot be generalized to higher age-groups. Very few studies reported on case management programmes in people older than 65 years and described the costs of intervention [12-14].

Thus, the aim of the present study was to evaluate the effect of a case management intervention by trained nurses on the time to first unplanned readmission or death in patients aged 65 years and older with myocardial infarction as compared with standard care.

## Methods

### Trial design and participants

KORINNA (“Coronary infarction follow-up in the elderly”) is a single-centre randomized two-armed parallel group trial of patients aged 75 years and older hospitalized with an acute myocardial infarction. The allocation ratio was 1:1.

Deviations from the study protocol include the decrease of minimum age of participants from 75 to 65 years, based on an unexpected low number of older aged persons which became apparent in the first year of recruitment. As a consequence, the study protocol was modified in coordination with the study’s Advisory Board by setting the minimum age of participants to 65 years. The recruitment phase was expanded accordingly. In addition, 11 persons (7 patients in the intervention group and 4 patients in the control group) were excluded because they died or withdrew consent before hospital discharge or did not meet the inclusion criteria. This deviation from the protocol was discussed with, and approved by the study’s Advisory Board.

Patients aged 65 and older who were hospitalized with a first or recurrent myocardial infarction between September 2008 and May 2010 in the Hospital of Augsburg, a tertiary care unit situated in the city of Augsburg, Southern Germany, were included consecutively. Patients who were in institutionalized care or planned to move into institutional care or outside the study region within the next months were excluded. Furthermore, patients with severe comorbidity (e.g. terminal cancer) which was associated with a life expectancy of < 1 year, and patients who were not able to communicate in German language were excluded. Finally, patients who were unable or unwilling to give written informed consent (e.g. patients with dementia) could not be included in the study.

The study protocol was approved by the Ethics Committee at the Bavarian Chamber of Physicians (Date of approval: 11.11.2008, Reference number: 08064). Furthermore, the study was conducted in accordance to German privacy law and in compliance with the Helsinki Declaration. The trial was registered in the Current Controlled Trials database in February 2009. Patients were recruited from 08.09.2008 to 11.05.2010 and one-year follow-up examinations took place from 29.09.2009 to 16.06.2011.

### Interventions

The nurse-based intervention is a complex intervention combining components from case-management and disease-management. Case-management elements include the identification of individual care problems and the facilitation of care coordination. Disease-management elements cover the identification of problems regarding management of cardiac risk factors and the provision of information and individual education. Main topics of the intervention were symptoms and management of heart failure (dyspnea, oedema, liquid control, body weight control), symptoms of angina, falls, blood pressure, heart rate, blood glucose, medication and medication adherence, depressed mood, and general physical condition. The selection of intervention’s components is based on the experiences with nurse-based follow-up programs in heart failure patients [15] and AMI patients [16]. It could be demonstrated that individual problem identification and corresponding care coordination and education can be effective measures to reduce readmission and death in CHD patients. Since elderly persons with AMI are more likely to have clinical and social risk factors that need specific consideration, it was expected that they benefit from an individual follow-up care.

Based on an assessment of these potential intervention areas using a structured interview guide, the study nurse estimated the need for an intervention and selected the type of intervention (e.g. referral to the general practitioner, individual education on medication use, contacts to cardiac sports group). For instance, if the patient reported that he/she fell down, the nurse may check the home environment for any trip hazards and may advise on its modification, may examine the patients’ walking abilities and advises on training or supportive devices, or may refer to the general practitioner in order to check possible adverse medication effects. The standards for conducting home visits and telephone calls, including potential interventions for each area, were provided in a standard operating procedures guide which has been developed by a multidisciplinary team consisting of the principal investigators, the study physician and the study nurses. The study nurses received a training on assessments and interventions by the study physician and the principle investigators. The intervention was tested and revised in a pilot phase from 27.06.2008 to 21.07.2008 in 11 patients. During the entire study course a team member joined selected intervention sessions as an observer to get a picture of the guideline adherence and the comparability of the applied interventions among the study nurses. Regular meetings among team members and study nurses were used to discuss problems regarding assessment and intervention and to enhance standardization.

The content and structure of home visits and telephone calls were comparable, and primarily differed regarding location and type of contact between the patient and nurse, and the possibility to perform for example blood glucose and blood pressure measurements.

**Figure 1:**
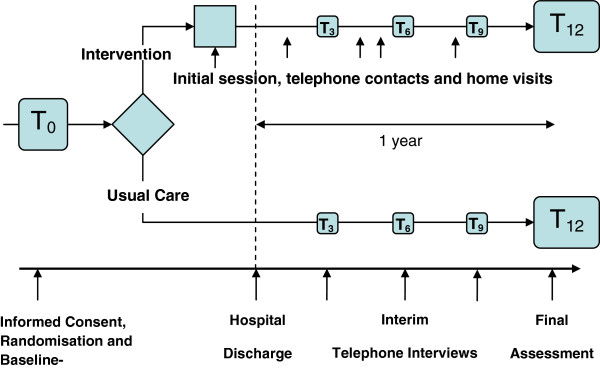
Study design.

The study design is shown in Figure 1. Briefly, after giving informed consent, all patients received a baseline assessment during hospital stay shortly before discharge, which was carried out by the study physician and a non-advanced practice study nurse. Those who were subsequently randomized to the intervention group received the intervention starting with the initial session shortly before discharge (for details see [17]).

In this session the patient was provided with information about the disease and comorbidities, about medication and with behavioural recommendations (nutrition, physical activity, smoking etc.). Information was given orally and in written form (so-called “heart book”). A first home visit was arranged 7 to 14 days after discharge, if accepted by the patient, otherwise an appointment for a telephone call was made. If the patient stayed in a rehabilitation hospital immediately after discharge from the Augsburg hospital, the first home visit was postponed accordingly.

Home visits (0 to 4) and telephone calls (at least every 3 months) were carried out according to patient need and patient risk level, which was assessed by the study nurse during the first home visit based on compliance, the social network, and the comorbidities. The risk level classification suggested by Russell et al. [18] was applied. Patients with New York Heart Association (NYHA) Classification class 1 or 2 who showed good compliance and social support were offered only telephone calls whereas for persons with limited compliance and/or social support at least one home visit was scheduled. Participants with NYHA classes 3 or 4 were planned to receive at least 2–3 home visits if they had limitations regarding compliance or social support or no impairments. In case patients with NYHA class 3 or 4 were lacking compliance and social support, at least 4 home visits were scheduled.

### Outcomes

The primary endpoint was measured as time between initial hospital discharge and first unplanned readmission to hospital or death. Readmission was defined as admission to any hospital after discharge from the index hospital. Only hospital stays with duration of at least 24 hours were included. In standardized non-interventional telephone interviews performed by study nurses with the patients in both groups at 3, 6, and 9 and 12 months after index hospital discharge patient’s readmissions, acute care visits to physicians, clinics, and ambulatory departments were identified. Self-reported readmissions were validated by hospital records and the study physician decided whether the readmissions were planned or unplanned ones. Blinding of the study physician was maintained. Outcome on all-cause mortality was determined from death certificates, which were obtained from the local health departments. After 12 months a follow-up examination was conducted.

The secondary endpoints of the KORINNA trial comprised clinical parameters such as blood pressure or lipid parameters, as well as functional capacity, nutritional risk, cognitive functioning, depression, and health-related quality of life. Functional ability was assessed using three different questionnaires: the Barthel Index [19], the Health Assessment Questionnaire (HAQ-DI) [20], and the Instrumental Activities of Daily Living Scale (IADL) [21]. Social support was assessed by using a questionnaire (F-sozU) [22], depressive symptoms were assessed by the Geriatric Depression Scale (GDS) [23], emotional well-being by the WHO-5 Well Being Index (WHO-5) [24], and cognitive function was measured by using the Mini Mental State Exam (MMSE) [25]. These instruments are described in more detail elsewhere [17]. The results are already submitted for publication elsewhere.

Another secondary outcome was the estimation of the cost-utility ratio of the case management intervention. Data for the economic analysis was collected by patient report and hospital records. Data on resource utilization was collected quarterly using a self- developed questionnaire based on available instruments such as the RAI (Resident Assessment Instrument) [26] or the RUD (Resource utilization of dementia) [27] and own experiences. Costs for the intervention excluding the costs caused by the study were calculated. Cost components included labor costs, travel expenses, telephone costs etc. In order to calculate quality-adjusted life years (QALYs), the EQ-5D questionnaire [28] was applied at baseline, at interim telephone contacts, and during the final assessment. Moreover, the Visual Analogue Scale (VAS) [28] was applied at baseline and final assessment. A cost-utility-analysis from the societal perspective including the incremental cost-effectiveness ratio (ICER) will be performed. More detailed information on the economic analysis is given elsewhere. The analysis is ongoing and results will be published elsewhere.

### Sample size

An event rate (readmission or death) of 40% in the control group was expected based on a comparable study with patients aged 70 years [16]. Our trial was designed to have at least 80% power to detect an improved rate of 25% in the intervention group (i.e. Δ = 0.15) at a two-sided type I error level of 5%. Thus, at least 152 patients per group were needed. We expected a drop-out rate not exceeding 10% during the 1-year follow-up period. In order to allow for loss to follow-up (patient withdrawing consent or moving away from study region), it was planned to recruit a total of 338 patients.

### Randomization

A randomization procedure using randomized blocks within strata was applied in order to achieve balanced treatment groups with respect to gender, age (< 70 vs. 70–79 vs. 80+), and number of comorbidities (diabetes and chronic heart failure). In order to ensure the concealment of the allocation, randomization was provided per telephone call to the biostatistical center at the Helmholtz Zentrum München where a randomization list was kept. Blinding of participants was not possible because home visits were only offered for participants from the intervention group. However, the persons who performed the final assessment after 1 year and the person who validated data on readmissions remained blinded towards the patient’s group assignment.

### Statistical methods

The primary analyses were conducted according to the intention-to-treat approach. Kaplan Meier curves were used to display the differences between the intervention and the control group regarding the time to event (i.e. till the first unplanned readmission or death). Cox proportional hazards regression was used to quantify the effect of intervention. For adjustment, the variables diabetes, heart failure, age and gender were included as independent variables into the Cox regression models. Subgroup analyses were performed for high risk groups (i.e. subjects aged 75 and above, subjects with diabetes, subjects with at least one comorbid condition) and for sex. As sensitivity analysis, the first six weeks were disregarded (i.e. the observation time started at day 42 after discharge), to approximate the time period since the first home visit.

## Results

**Figure 2:**
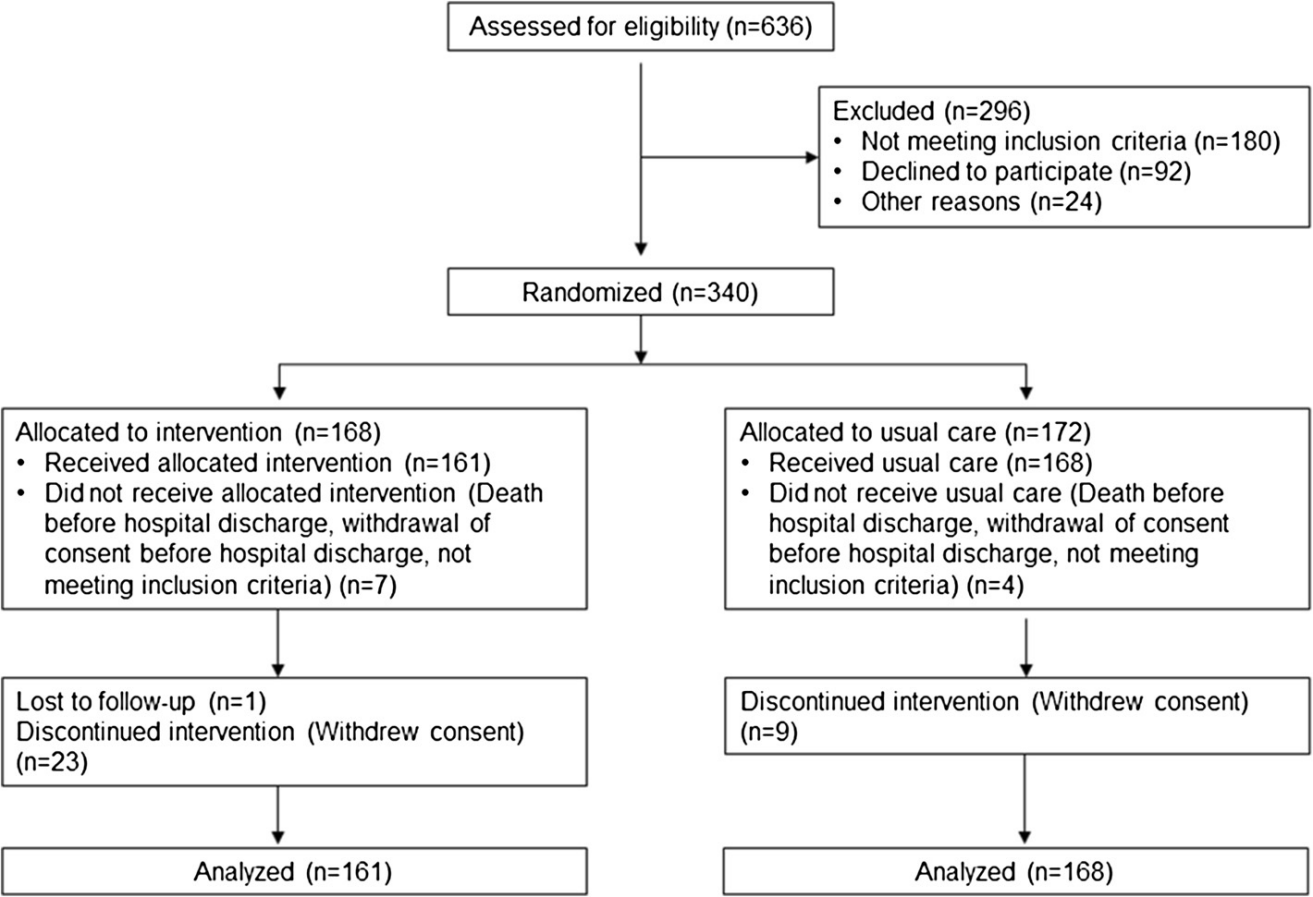
Flow of participants through the KORINNA trial.

In all, 636 patients were screened for participation in the study. Of those, 296 patients were not included as they did not wish to participate (n=92), did not fulfill the inclusion criteria (n=180) or met other exclusion criteria (n=24). Thus, 340 patients were included and underwent randomization to follow-up for one year within the nurse-based management (intervention group) or to usual care (control group). After randomization, 11 persons (7 patients in the intervention group and 4 patients in the control group) were excluded because they died or withdrew consent before hospital discharge or did not meet the inclusion criteria. Thus, 161 persons started the allocated intervention and 168 individuals received usual care (see Figure 2).

### Baseline characteristics

The baseline characteristics of the 329 participating patients are shown in Table 1. The two groups were well matched for gender and age. The mean age of the overall sample was 75.4 (± 6.0) years. In the intervention group, 29.8% of the patients suffered from heart failure and 28.0% had diabetes; the corresponding percentages in the control group were 28.0% and 36.3%. Intervention and usual care patients did not differ regarding the scores, mean BMI and diastolic blood pressure at baseline examination. The mean systolic blood pressure was 121.6 mmHg in the intervention group in comparison to 124.2 mmHg in the control group.

**Table 1:** Patient characteristics at baseline

**Characteristic**	**Intervention group (n=161)**	**Control group (n=168)**
**Sociodemographics and comorbidities**		
Mean age, years (SD)	75.2 (6.0)	75.6 (6.0)
Male sex, % (n)	62.7% (101)	61.3% (103)
Diabetes mellitus, % (n)	28.0% (45)	36.3% (61)
Congestive heart failure, % (n)	29.8% (48)	28.0% (47)
**Blood pressure,** mean (SD)*		
Systolic BP, mmHg	121.6 (13.7)	124.2 (13.5)
Diastolic BP, mmHg	71.4 (7.8)	71.3 (8.3)
**BMI (kg/m**^**2**^**),** mean (SD)*	27.7 (4.2)	27.3 (3.9)
**Physical/mental health,** mean (SD)*		
HAQ-DI score	0.8 (0.8)	0.8 (0.8)
Barthel-index	90.8 (17.1)	90.8 (17.5)
IADL-Score	5.6 (1.6)	5.6 (1.5)
MMST	26.7 (4.1)	26.4 (3.8)
GDS	3.2 (3.1)	3.2 (2.6)
Social support	3.9 (0.6)	3.9 (0.6)
WHO-5-well-being index	13.6 (6.8)	13.2 (6.9)

*Due to missing values, the number of observations ranges between 155 and 161 in the intervention, and 161 and 167 in the control group.

Altogether 132 of the 161 patients (82%) in the intervention group accepted a first home visit; during the follow-up period of one year 186 home visits were carried out (104 patients received 1 visit, 10 patients received 2 visits, 11 patients received 3 visits, 6 patients received 4 visits, and 1 patient received 5 home visits). A home visit lasted 117 minutes on average. Furthermore, in the intervention group, 489 telephone appointments were made, that is, 161 patients received on average 3 telephone appointments. The mean length of a telephone session was 19 minutes. Topics of the discussions with the patients in the intervention group were symptoms and management of heart failure, symptoms of angina, falls, control of blood pressure, heart rate, and blood glucose, medication and medication adherence, depressed mood, and general physical condition.

### Observed events

The mean follow-up time for the intervention group was 273.6 days, and for the control group it was 320.6 days. During the follow-up period, 140 patients had an event (i.e. first unplanned readmission or death). Sixty-two of these were in the intervention group and 78 in the control group. In the intervention group 57 of the events were first unplanned readmissions and 5 were deaths, while in the control group 75 of the events were first unplanned readmissions and 3 were deaths. Furthermore, 13 patients in the intervention group and 10 patients in the control group of those with unplanned readmissions died within the follow-up period.

Many events appeared within the first two months after discharge. Within the first 60 days after discharge there were 34 events in the intervention group and 28 events within the control group. Of these 29 events in the intervention group and 27 events in the control group were unplanned readmissions and 5 in the intervention group and 1 in the control group were deaths. Furthermore, 10 patients in the intervention group and 5 patients in the control group of those with unplanned readmissions died within the first 60 days after discharge.

During the follow-up period altogether 235 unplanned admissions and 79 planned admissions to hospitals occurred in the whole study group. Of those, there were 87 unplanned and 46 planned hospital stays in the intervention group, and 148 unplanned and 33 planned hospital stays in the control group. Eighty patients in the control group and 85 patients in the intervention group had no hospital stay during the one-year follow-up.

Figure 2 shows the time-to-event Kaplan-Meier-Curves for the combined end point of unplanned readmissions and death in the two study groups. No significant beneficial effect for the intervention group in comparison to the control group could be shown after one year (Hazard Ratio 1.01, 95% confidence interval 0.72-1.41, Table 2). However, the Kaplan Meier curves show a crossing which favors the intervention group at the end of the follow-up period.

**Table 2:** Results of the Cox regression model

**Parameter**	**Estimate**	**Standard error**	**Hazard ratio**	**95% confidence interval**	**P value**
Intervention group	0.07	0.17	1.01	0.72; 1.41	0.969
Diabetes	0.38	0.18	1.46	1.02; 2.09	0.039
Heart failure	0.35	0.19	1.42	0.99; 2.04	0.060
Age	0.06	0.02	1.06	1.03; 1.10	<.001
Male	−0.21	0.19	0.81	0.56; 1.17	0.264

### Subgroup analysis

**Figure 3:**
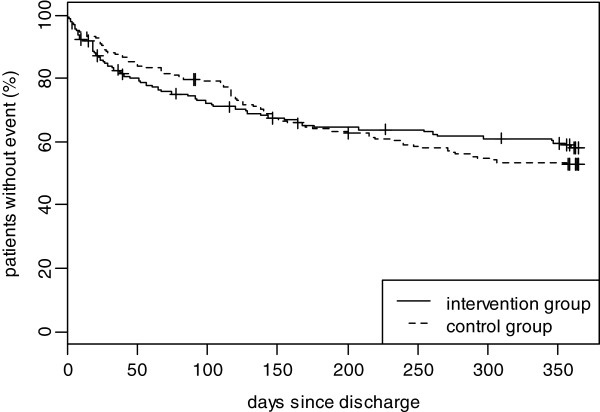
Kaplan Meier curve until first unplanned readmission to hospital or death (total sample).

**Figure 4:**
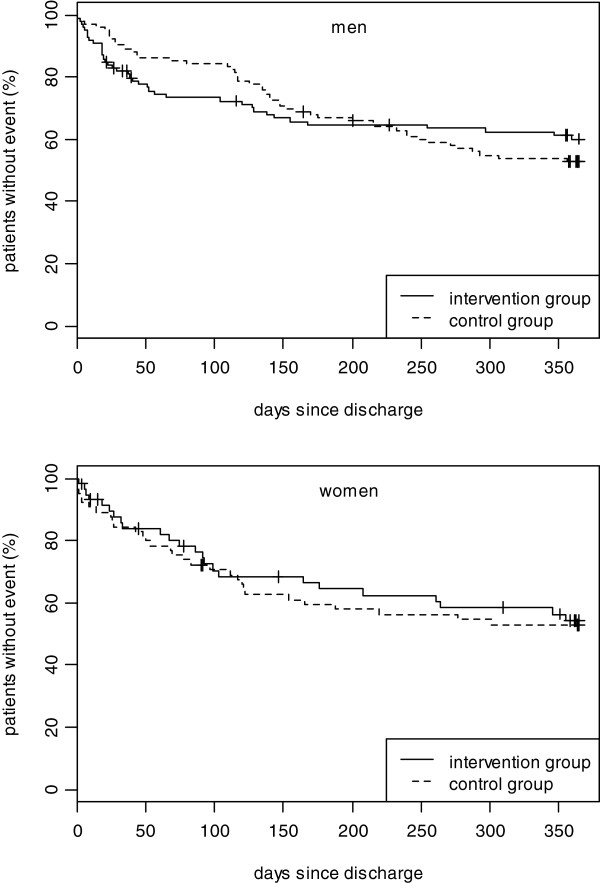
top: Kaplan Meier curve until first unplanned readmission to hospital or death (men), bottom: Kaplan Meier curve until first unplanned readmission to hospital or death (women).

Figure 3 shows the survival curves for the combined end point for the whole study group and Figure 4 shows the survival curves stratified by sex. After one year, neither in men nor in women significant differences between the intervention and the control group could be observed. In male participants the same pattern with a cross in favour of the intervention group could be observed (Figure 4 top). However, in women the intervention group seems to have a beneficial but insignificant effect right from the start (Figure 4 bottom).

No significant beneficial effects of the intervention on the end point unplanned readmissions and death were consistently found in further subgroup analyses. While the crossing in favour of the intervention group could be observed among the subgroup of diabetic persons, persons living alone, and persons with at least one comorbid condition, in the subgroup analysis including patients aged > 75 years this pattern was not found (data not shown).

### Sensitivity analysis

As sensitivity analysis, the first six weeks were disregarded, to approximate the time period since the first home visit. In this analysis adjusted for diabetes, heart failure, age, and gender, regarding the time to first unplanned hospitalization or death also no significant beneficial effect for the intervention group in comparison to the control group could be shown after one year (Hazard Ratio 0.90, 95% confidence interval 0.62-1.29, p-value 0.556).

## Discussion

In the present study, no significant beneficial effect of a case management intervention by trained nurses on the time to first unplanned readmission or death within one year after discharge in patients 65 years and older with myocardial infarction as compared with standard care could be shown. Furthermore, in subgroup analysis no patient group with a significant benefit from the intervention could be identified. However, because the Kaplan Meier curves show a crossing in favor of the intervention group during follow-up a delayed positive effect of the case management intervention by trained nurses might be possible.

So far, a lot of intervention trials investigated the effect of a nurse-based case management on the frequency of readmissions and other outcomes in persons with CHD [12,13]. In a meta-analysis including 63 randomized trials by Clark et al. the impact of secondary prevention programs with and without exercise components in patients with CHD predominantly younger than 70 years were investigated [12]. It could be shown that secondary prevention programs have a positive effect on process of care and functional status or quality of life and reduce MIs. The found mortality benefit became stronger with longer follow-up (24 months) and the advantages were independent of the key components of the respective interventions.

In a review including 12 randomized trials conducted in persons mainly younger than 70 years the effect of case management programmes for patients with established CHD on the process of care and mortality was examined [13]. It was found that multidisciplinary disease management programmes for secondary prevention have a beneficial effect on process of care in patients with CHD. With the case management programmes it was possible to significantly reduce admissions to hospital and to improve quality of life. However, these randomized clinical trials failed to show any survival benefit or reduction in recurrent MI. Furthermore, the duration of intervention and length of follow-up had no effect on the observed results.

The results of these studies are promising, but the findings cannot be generalized to higher age-groups. Only very few studies investigating the benefit of case management programmes included persons older than 65 years. For example, Naylor et al. [14] examined whether a comprehensive discharge planning and home follow-up intervention in elderly hospitalized persons (mean age 75 years) at high risk for rehospitalisation by advanced practice nurses has an effect on outcomes. It could be demonstrated that such an intervention can reduce readmissions, lengthen the time between discharge and readmission, and decrease the costs of providing health care in older patients with one of several medical and surgical reasons for admission [14].

In the KORINNA study older patients with myocardial infarction, who have often been excluded from randomized controlled trials were included. Contrary to the results of most of the prior studies we could not demonstrate a significant effect of the nurse-based intervention on the primary outcome during the one year follow-up in this patient group. The reasons for a missing significant benefit for the persons managed in the intervention group are not clear. Possibly, the study period of 12 months was too short to show a clear impact on readmissions and mortality. This assumption can be corroborated by the fact that the survival curves cross during follow-up in favour of the intervention group, which could point to a delayed effect of the case management by study nurses. As an effect of the nursing intervention one could imagine that patients first become more sensitive to potential harmful changes of their health status which might explain the observed higher number of hospital readmissions in the first four months of the study period. This increased awareness, however, might be associated with a higher treatment adherence that could positively affect long-term outcomes. At the moment it is unclear whether there could be a significantly positive long-term intervention effect on readmission to hospital or death in the present study. However, we were able to get additional funding for an extension and data from the 3-year follow-up will be used to further elucidate this question.

In addition, it is not clear whether the timing of the interventions was optimal to increase their effectiveness. Perhaps more frequent interventions in the first two months would be more useful. However, systematic reviews so far could not identify intervention schedules which were particularly effective, thus, further research is warranted [13,29].

Of interest, women assigned to the intervention group, showed a tendency for less hospital readmissions and better survival compared with the control group from the beginning of the study whereas in men the typical cross of survival curves was found. Considering results from studies which indicate that gender-specific secondary prevention programs are more effective than unspecific ones [30], one can speculate that the intervention applied in the KORINNA study may include modules which have a greater short-term benefit in women than in men. Thus, it is essential that a more detailed analysis of the differential impact of the intervention program will be performed. Moreover, it is thinkable, that disease management programmes may be most beneficial in those settings where usual care is suboptimal. Hence, it could be assumed, that in the present study the incremental benefit of the nurse-based management over the usual care group may be very small because the management in the usual care arm may be close to optimal already. Furthermore, another explanation for the missing benefit for the intervention group might be that on the patient level the intervention had no significant, sustainable impact on the behaviour of the patients. In addition, it is thinkable that our study is not comparable to other randomized controlled trials on this issue conducted in other countries or settings due to differences in the kind of intervention, standard care and study endpoints. In a systematic review including twenty-one randomized clinical trials of transitional care interventions targeting chronically ill adults Naylor et al. found a substantial heterogeneity in the populations, settings, interventions and methodologies, which could influence the study results. The authors of that study have identified three proven strategies that have effectively reduced all-cause readmissions through six of twelve months. It was concluded that many of the successful interventions shared similar features and recommended several strategies to guide the implementation of transitional care [31]. Olson et al. found in their systematic review including 62 articles representing 44 studies that most studies did not consistently demonstrate that any specific intervention resulted in improved patient- or system-based outcomes. Thus, more consensus is needed on the definition of the interventions and the outcomes appropriate to those interventions [32].

A possible limitation of the study design is the relatively short follow-up interval of one year. Hospital readmissions were determined based on patient self-reports and may be biased. The generalizability of our results is limited to elderly patients with a myocardial infarction and may not apply to other populations. A further limitation of the study is its single center design. Furthermore, only 340 out of the 636 patients assessed for eligibility participated in the study, 21% (92 of 432) declined to participate prior to randomization, and another 10% (34 of 331) withdrew consent after randomization. If there were systematic differences between the excluded patients and those randomized to the trial, the external validity of the present findings may be reduced.

## Conclusions

In summary, the KORINNA study showed that a nurse-based management over one year did not seem to significantly influence unplanned readmissions to hospital or out of hospital deaths in comparison to usual care in older myocardial infarction patients. Whether such an intervention could have a long-term influence on these outcomes should be the focus of further studies.

## Competing interests

The authors declare that they have no competing interests.

## Authors’ contributions

CM, BK and RH initiated the study and raised the research grant. CM and BK are principal clinical investigators, RH is principal statistical investigator. CM and BK developed the case-management programme. CM, RH, BK, RW, HS contributed to conception and design of the study. HS is responsible for the data monitoring. All authors contributed to monitoring and conducting the study. BS conducted the statistical analysis. RW is study physician and was responsible for patient recruitment and clinical examination/assessment. IK is in charge of the local coordination of the study. All authors critically revised and approved the final manuscript.

## Pre-publication history

The pre-publication history for this paper can be accessed here:

http://www.biomedcentral.com/1471-2318/13/115/prepub
